# Marennine, Promising Blue Pigments from a Widespread Haslea Diatom Species Complex

**DOI:** 10.3390/md12063161

**Published:** 2014-05-28

**Authors:** Romain Gastineau, François Turcotte, Jean-Bernard Pouvreau, Michèle Morançais, Joël Fleurence, Eko Windarto, Fiddy Semba Prasetiya, Sulastri Arsad, Pascal Jaouen, Mathieu Babin, Laurence Coiffard, Céline Couteau, Jean-François Bardeau, Boris Jacquette, Vincent Leignel, Yann Hardivillier, Isabelle Marcotte, Nathalie Bourgougnon, Réjean Tremblay, Jean-Sébastien Deschênes, Hope Badawy, Pamela Pasetto, Nikolai Davidovich, Gert Hansen, Jens Dittmer, Jean-Luc Mouget

**Affiliations:** 1 FR CNRS 3473 IUML, Mer-Molécules-Santé (MMS), Université du Maine, Ave O. Messiaen, 72085 Le Mans cedex 9, France; E-Mails: gastineauromain@yahoo.fr (R.G.); eko_windarto89@yahoo.co.id (E.W.); fsembapr@gmail.com (F.S.P.); sulastriarsad@yahoo.co.id (S.A.); Boris.Jacquette@univ-lemans.fr (B.J.); Vincent.Leignel@univ-lemans.fr (V.L.); Yann.Hardivillier@univ-lemans.fr (Y.H.); 2 Institut des Sciences de la mer de Rimouski, Université du Québec à Rimouski, 310 des Ursulines, Rimouski, QC G5L 3A1, Canada; E-Mails: francois.turcot@gmail.com (F.T.); mathieu_babin@uqar.ca (M.B.); Rejean_Tremblay@uqar.ca (R.T.); jean-sebastien_deschenes@uqar.ca (J.-S.D.); 3 EA 1157, Laboratoire de Biologie et Pathologie Végétales (LBPV), Université de Nantes, SFR 4207 QUASAV, 44322 Nantes, France; E-Mail: Jean-Bernard.Pouvreau@univ-nantes.fr; 4 FR CNRS 3473 IUML, Mer-Molécules-Santé (MMS), Université de Nantes, 44322 Nantes, France; E-Mails: michele.morancais@univ-nantes.fr (M.M.); Joel.fleurence@univ-nantes.fr (J.F.); laurence.coiffard@univ-nantes.fr (L.C.); celine.couteau@univ-nantes.fr (C.C.); 5 FR CNRS 3473 IUML, UMR-CNRS 6144 (GEPEA), Université de Nantes, CRTT 37 Bd de l’Université, F-44602 Saint-Nazaire, France; E-Mail: pascal.jaouen@univ-nantes.fr; 6 UMR CNRS 6283, Institut des Molécules et Matériaux du Mans (IMMM), Université du Maine, Ave O. Messiaen, 72085 Le Mans cedex 9, France; E-Mails: Jean-Francois.Bardeau@univ-lemans.fr (J.-F.B.); hope.badawy@gmail.com (H.B.); Pamela.Pasetto@univ-lemans.fr (P.P.); Jens.Dittmer@univ-lemans.fr (J.D.); 7 Department of Chemistry, Université du Québec à Montréal, P.O. Box 8888, Downtown Station, Montréal, QC H3C 3P8, Canada; E-Mail: marcotte.isabelle@uqam.ca; 8 Université de Bretagne-Sud, Laboratoire de Biotechnologie et Chimie Marines, Campus de Tohannic, F-56017 Vannes, France; E-Mail: nathalie.bourgougnon@univ-ubs.fr; 9 Karadag Nature Reserve of the National Academy of Sciences, p/o Kurortnoe, Feodosiya 98188, Crimea; E-Mail: NickolaiD@yandex.ru; 10 Department of Biology, Marine Biological Section, University of Copenhagen, Øster Farimagsgade 2D, 1353 Copenhagen, Denmark; E-Mail: gerth@bio.ku.dk

**Keywords:** aquaculture, biological activities, cosmetics and food industry, *Haslea ostrearia*-like diatoms, marennine-like blue pigments

## Abstract

In diatoms, the main photosynthetic pigments are chlorophylls *a* and *c*, fucoxanthin, diadinoxanthin and diatoxanthin. The marine pennate diatom *Haslea ostrearia* has long been known for producing, in addition to these generic pigments, a water-soluble blue pigment, marennine. This pigment, responsible for the greening of oysters in western France, presents different biological activities: allelopathic, antioxidant, antibacterial, antiviral, and growth-inhibiting. A method to extract and purify marennine has been developed, but its chemical structure could hitherto not be resolved. For decades, *H. ostrearia* was the only organism known to produce marennine, and can be found worldwide. Our knowledge about *H. ostrearia-*like diatom biodiversity has recently been extended with the discovery of several new species of blue diatoms, the recently described *H. karadagensis*, *H. silbo* sp. inedit. and *H. provincialis* sp. inedit. These blue diatoms produce different marennine-like pigments, which belong to the same chemical family and present similar biological activities. Aside from being a potential source of natural blue pigments, *H. ostrearia-*like diatoms thus present a commercial potential for aquaculture, cosmetics, food and health industries.

## 1. Introduction

Seas and oceans cover more than 70% of the Earth’s surface; water mainly transmits and scatters blue wavelengths while absorbing the red part of the solar light spectrum, thus leading astronauts to say our planet is blue. In or under the sea, however, the blue color is scarcely distributed, as it can be observed only in a few organisms, such as the blue jellyfish *Cyanea lamarckii*, the blue coral *Heliopora coerulea*, the blue sea star *Linckia laevigata*, the giant clam *Tridacna maxima*—the mantle of which is commonly bright blue—and some species of surgeonfish from the genus *Acanthurus*, all of which being emblematic species. Micro-organisms appear to be better providers of the blue color, as blue pigments have been evidenced long ago among bacteria, albeit first in terrestrial species. For instance, in Proteobacteria, *Pseudomonas aeruginosa* produces pyocyanin [1,2], a blue pigment that possesses antibiotic activities [3], and *Pantoea agglomerans* has recently been shown to produce a novel “deep blue” pigment [4]. The study of marine bacteria started and expanded later, but a few species are known today to synthesize blue pigments [5]. For example, glaukothalin is produced by different species from the genus *Rheinheimera* [6,7], and indigoidine is encountered in a strain of the marine bacteria genus *Phaeobacter*, in which it plays a role in the colonization of surfaces [8]. It is worth noting that indigoidine is also encountered in the terrestrial plant pathogen *Erwinia chrysanthemi* [9], in which this blue pigment seems to be partly responsible for its pathogenicity [10].

Regarding photosynthetic organisms, blue pigments can be observed in prokaryote as well as in eukaryote species. Formerly known as “blue algae”, cyanobacteria have specific accessory protein–pigment complexes, the phycobiliproteins. Some of these phycobiliproteins, like phycocyanin and allophycocyanin, have a blue color due to their absorption of orange and/or red light [11]. Phycobiliproteins were discovered in the 19th century, phycocyanin being first described in a strain of *Oscillaria* sp. [12], and allophycocyanin in the red macroalga *Porphyra vulgaris* [13]. The partial protein nature of these two molecules was suggested by Mölisch [14], in his work on phycoerythrin [15]. Kylin [16] further demonstrated that each molecule was a complex of a chromophore (phycobilin) covalently bound to a protein. Phycocyanin and allophycocyanin are not restricted to cyanobacteria, as they have been evidenced in two groups of photosynthetic eukaryotes, Rhodophyta and Glaucophyta. Members of another group, the Cryptophyta, contain only one of these two blue pigments, phycocyanin (e.g., [17]). Until very recently, only two other photosynthetic eukaryotes, both members of the Heterokontophyta, were known to produce blue pigments, the recently discovered *Aurearena cruciata* (Aurearenophyceae) in its senescent stage [18], and the long-known pennate diatom *Haslea ostrearia* (Bacillariophyceae), during its exponential phase of growth and aging [19]. *H. ostrearia*, the “blue navicula,” produces the specific pigment marennine, responsible for the “greening” of oysters. 

In this review, we focus on the diatom *H. ostrearia*, its blue pigment marennine, and their many interactions with oysters. We present an historical perspective of our knowledge of marennine complemented by some new insights, regarding its chemical nature, characterization, and biological properties, particularly considering biophysical processes required to absorb light. Indeed, an explanation why the color blue is relatively seldom in organisms and organic molecules could be related to the physical processes required to absorb red or yellow light, that is, light with relatively low energy. If the color is not stemming from metal centers, it is usually the excitation of delocalized electrons from their molecular orbital ground state π to the excited state π*, which makes a molecule appear colored. One double bond alone is usually not sufficient as it absorbs light in the UV range. Only the conjugation of several double bonds broadens the energy range of the different π orbitals, reducing the energy gap between the highest π and the lowest π* orbital. More than ten conjugated double bonds are necessary to reduce the energy gap to absorb red light [20] so that the molecule appears blue, and this is a rather rare constellation. Similar considerations count for aromatic rings, where usually adjacent groups can reduce the absorption energy, but hardly into the red region. 

We also present data about *H. karadagensis*, the second species of blue diatoms recently discovered in the Black Sea, and *H. provincialis* sp. inedit. and *H. silbo* sp. inedit*.*, two new blue diatom species discovered in the Mediterranean Sea and the Canary Islands, respectively [21]. At least one of the blue pigments produced by these new species (*H. karadagensis*) is different from marennine, but all belong to the same chemical family. Finally, we address the unexpected biodiversity of blue diatoms, and the added-value potential of marennine-like pigments, in aquaculture, cosmetics, food and health industry. 

## 2. The Marennine–Oyster Connection

**Figure 1:**
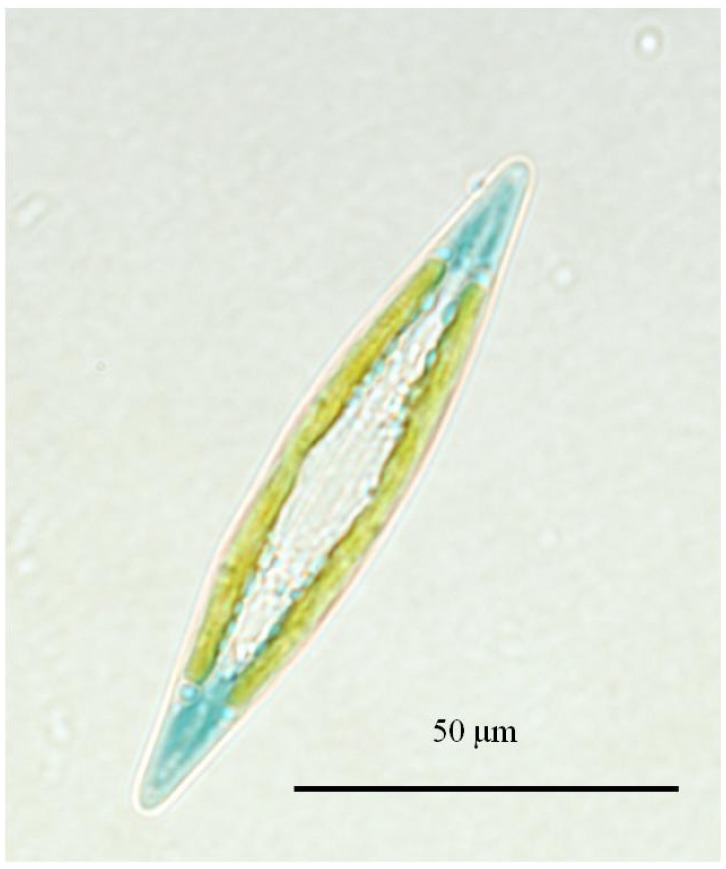
Light micrograph of *Haslea ostrearia*.

In the following, we will refer to blue pigments as “marennine” when produced by *H. ostrearia*, and as “marennine-like pigments” when produced by another species of blue diatoms (Figure 1). Indeed, no other name can be provided in accordance with the IUPAC standards, as the chemical structures of these pigments are as of yet unresolved. Marennine is the anglicized version of “marennin,” proposed by Lankester [22], in reference to Marennes-Oléron (Marennes, 45°49′25.8″ N 1°06′16.3″ W), a region of western France where oysters have been farmed for centuries, especially renowned for producing “green oysters”. In the Marennes-Oléron Bay, as well as in Bourgneuf Bay (Bouin, 46°58′27.4″ N 1°59′54.9″ W), oysters are matured and fattened in old salt marshes, the so-called “claires”. In these shallow and nutrient-rich ponds, phytoplankton proliferates and may feed oysters whose size rapidly increases and whose organoleptic properties improve. The duration of the fattening period and the density of the oysters in the ponds are regulated according to specific standards for the bivalves to be commercialized as “fines de claires.” In these ponds, *H. ostrearia* may become dominant year after year, producing large amounts of marennine, which are released into the seawater. It adheres to oyster gills, a phenomenon that increases culinary attractiveness and therefore the market value of the bivalves (red label “fines de claires vertes”). 

### 2.1. A Brief History of the Greening of Oysters, Signature of the Diatom Haslea ostrearia

As a biological phenomenon, green oysters provoked questions and wonderment for centuries. The earliest indication known is “The history of the generation and ordering green oysters, commonly called Colchester-oysters”, by Thomas Sprat, at the end of the 17th century [23]. Thomas Sprat was an Anglican bishop of Rochester, famous for his *History of the Royal Society of London*, in which he included an article on the green oysters of Colchester. He mentioned that oysters from the Tolesbury ponds could present a green color during summer, a phenomenon he suspected resulted from the actions of the sun and the Earth, leading to a green coloration of pond bottom, before oyster gills turn green. The same environmental causal factors, along with the brackishness of waters, were addressed 150 years later [24]. Other hypotheses, not referring to environmental issues, ascribed the greening to a disease similar to obesity [25], or to “liver malfunction” [26]. The role of grass, plants and mosses grown on the shores of the ponds and possibly ingested by oysters or some kind of Priestley’s green matter was also hypothesized [27,28,29]. In line with these speculations, some authors suspected that the water of ponds, and consequently oysters, could be colored by pigments originating from green macroalgae [30]. Finally, some authors estimated that greening could result from the presence of specific metallic ions in pond sediments [31], or from unusual interactions with copper [32,33], a metal long known for inducing changes in color in oysters [34]. Indeed, such unusual color may be perceived as unappetizing, possibly reflecting the presence of pollutants as zinc or copper [35,36,37]. In France, however, green oysters are gastronomically famous and more expensive than ordinary oysters. Moreover, from an historical perspective, green oysters have always been celebrated in this country as a delicacy [38,39,40], even being considered a dish fit for a king—at the very least for the Sun King; it was one of Louis XIV’s favorite meals [41].

The first experimental work on green oysters was reported by Benjamin Gaillon [42], an officer of French Customs during the Restoration and the early July Monarchy. Gaillon worked in the town of Dieppe (Normandy) and dedicated his free time to the life sciences, with a special interest in green oysters, common at that time in the Dieppe area. Gaillon sampled green oysters, scraped their shells and made microscopic examinations. He observed small motile organisms with a “green” color, not blue like *H. ostrearia* cells, possibly because of the limits of his microscope device. Gaillon hypothesized that these motile organisms could be responsible for the greening of oysters, and considered them to be animals, which he called *Vibrio ostrearius*, based on the classification of worms by Bruguière [43]. Controversially but not impartially, Goubeau de la Bilennerie [24] argued that the greening exclusively depended on environmental factors and oyster farmers’ know-how, a phenomenon thus appearing more acceptable for oyster consumers. Indeed, a possible impact of Gaillon’s theory on consumers and green oyster sales was of some concern for Goubeau de la Bilennerie, president of the court in Marennes (for a full study, see [44]).

### 2.2. Haslea ostrearia, a Very Uncommon Diatom

The topic of green oysters involved Gaillon in a second controversy, with Jean-Baptiste Bory de Saint-Vincent, botanist, explorer, and Dragoons cavalry officer during the Napoleonic wars. Whereas Gaillon advocated for an animal nature of *V. ostrearius*, Bory de Saint Vincent classified it alongside diatoms into his “psychodiaire” kingdom, which contained all organisms whose position between animal and plant was unclear. Bory [45] thus proposed the name *Navicula ostrearia*, which remained for 150 years. Taking advantage of scanning electron microscopy facilities, Simonsen [46] transferred the “blue navicula” from the genus *Navicula* to *Haslea*, a new genus he created for this purpose, based on specific morphological features of the frustule, and he used *H. ostrearia* as the type-species. By doing so, Simonsen acknowledged G.R. Hasle for her considerable work on phytoplankton in general, and on diatoms in particular. 

**Figure 2:**
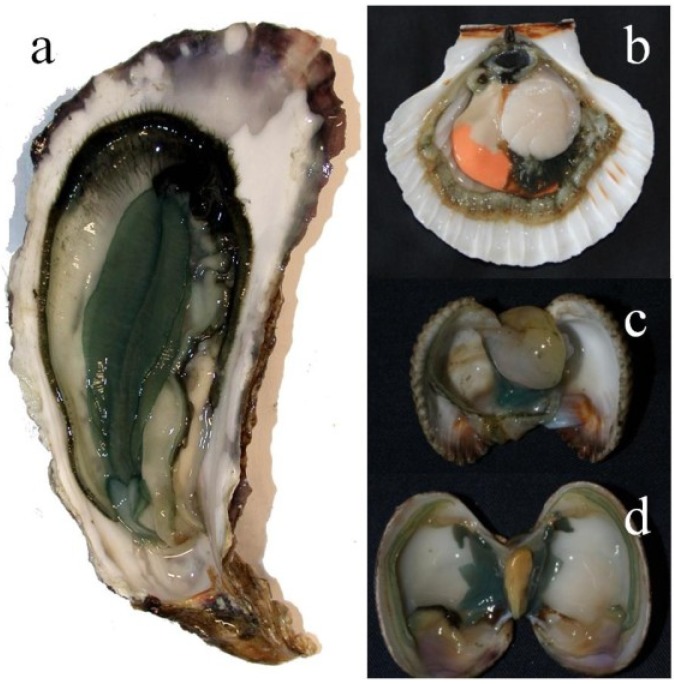
Greening effect of marennine on bivalves. (**a**) Pacific oyster; (**b**) Scallop; (**c**) Cockle; (**d**) Clam.

*Haslea ostrearia* is a tychopelagic diatom [47]—an organism that can be benthic or epiphyte—but also planktonic [48]. *H. ostrearia* is euryhaline [49,50], and can develop in high light environments [51]. Thus this diatom seems well adapted to oyster ponds, characterized by shallow and nutrient-rich water, where it mainly proliferates in autumn/spring, and can outcompete other microalgae [47,52]. Marennine produced during *H. ostrearia* blooms is released into the seawater, and the ponds turn green. In such ponds, oysters can become green in a few days, by exhibiting light to dark-green gills (Figure 2a). This phenomenon is not restricted to oyster ponds in western France, as it can happen spontaneously elsewhere, in Great Britain [23], Denmark [53], the United States [36,54], *etc*. It is worth noting that the greening happens naturally in peculiar environments, usually protected bays with quiet waters and fresh water inlets. The greening is not limited to oysters, as it also occurs in other invertebrates, polychaetes, crabs, littorina, mussels [55], sea anemones [42], scallops, clams and cockles (Figure 2b–d). If greening invertebrates is not the blue diatoms’ *raison d’être*, it is as least their signature. 

### 2.3.An Expanding Species—Complex of Blue Diatoms from the Genus Haslea

**Figure 3:**
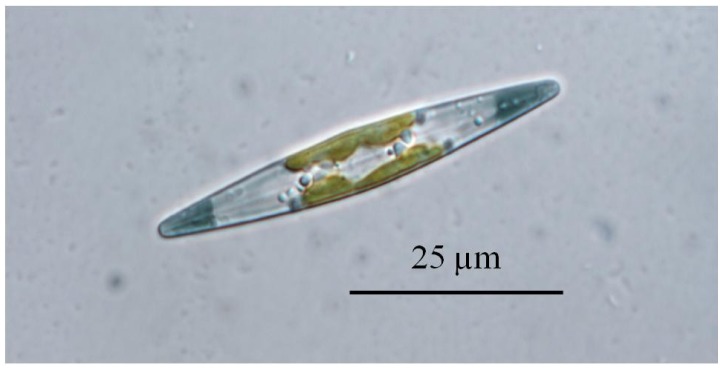
Light micrograph of *Haslea karadagensis*.

Over the past two centuries, diatoms with blue tips, or marine invertebrates with greened gills have been reported from almost all seas and oceans, which made *Navicula* or *Haslea ostrearia* a unique and cosmopolitan species [56,57], and marennine a curiosity. Indeed, all blue diatoms observed were ascribed to *N.* or *H. ostrearia*, with the possible exception of a sample collected in Honduras, identified as *N. fusiformis* var. *ostrearia* by Grunow [58]. The biodiversity of blue diatoms has recently been unraveled, with the collection of phytoplankton samples in different countries and continents, and their examination using various and complementary methods, *i.e*., biometry of natural populations, morphometry of the frustules, molecular markers, reproductive compatibility, UV-visible spectrophotometry. The first novelty came from the shores of the Black Sea (Karadag Biological Station, Crimea) with a second species of blue diatom characterized and named *Haslea karadagensis* [56]. Despite a similarity with *H. ostrearia* when observed in light microscopy, striae density of the frustule [56], *rbc*L (RubisCO large sub-unit) and ITS (Internal Transcribed Spacers) markers [56], and the inability to interbreed [59], constitute evidence that they are two different species. Another major difference that allows distinguishing between the two species concerns their pigments, as blue apices in *H. karadagensis* appear darker than in *H. ostrearia* (Figure 3). Both pigments exhibit a comparable and reversible bathochromic shift when pH increases, but different λ_max_ [21]. Furthermore, UV-visible spectrophotometry shows that the pigment produced by *H. karadagensis* presents two isobestic points when pH varies from 2 to 12 [21], in comparison with only one in marennine [21]. Regarding biological properties, both pigments demonstrated a greening effect on bivalves, and as detailed below, antibacterial, antiviral and antifungal activities [57,60]. 

**Figure 4:**
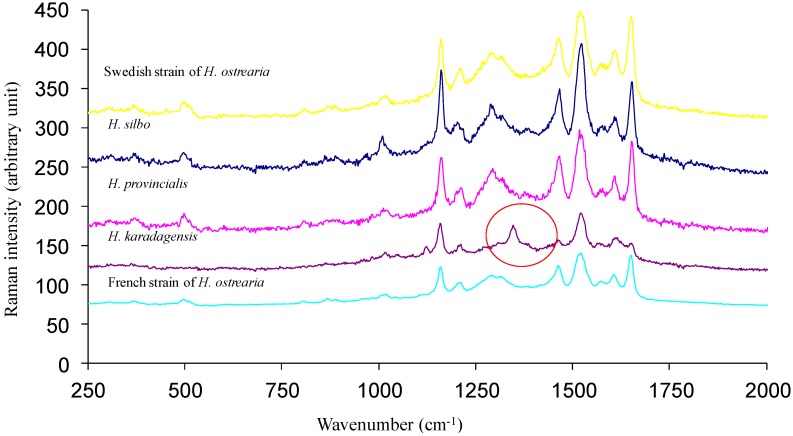
Raman spectra obtained *in vivo* on the blue pigments contained in the apices of different strains of blue diatoms. Noticeable differences can be seen between the pigment of *H. karadagensis* and the others in the 1240 cm^−1^ to 1420 cm^−1^ region.

**Figure 5:**
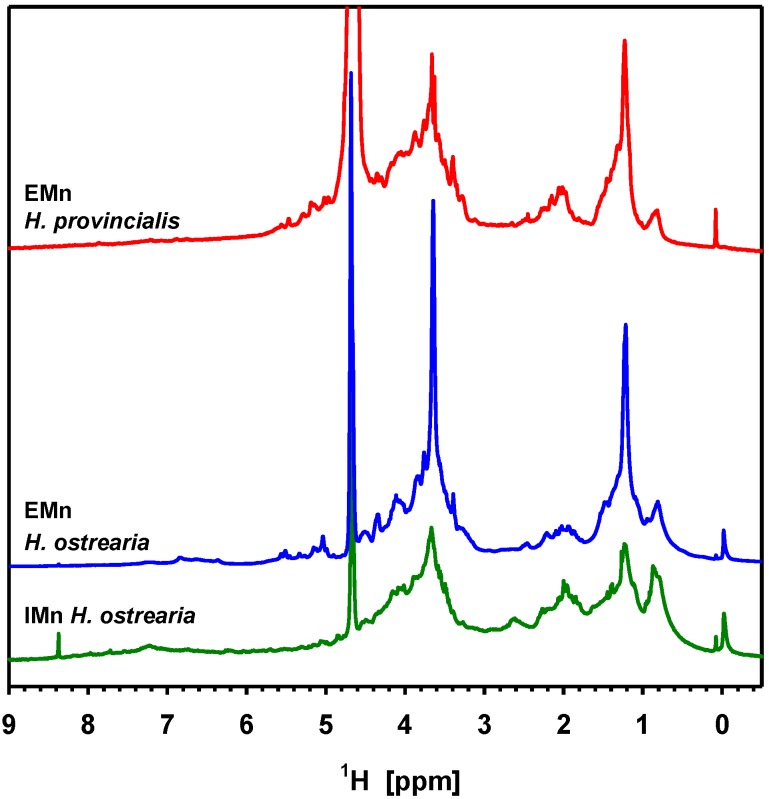
[1H NMR]. ^1^H 1D NMR spectra of the intracellular form (IMn) of marennine from *Haslea ostrearia* (bottom, green), the extracellular form (EMn) of marennine (middle, blue), and the extracellular form of the marennine-like pigment from *Haslea provincialis* sp. inedit*.* (top, red), all dissolved in D_2_O. The signal at 4.7 ppm stems from residual HDO, the high signal at 3.64 ppm in the extracellular marennine partly stems from an impurity.

More samples of blue diatoms were obtained from the Canary Islands (La Gomera), and the Mediterranean Sea (French coast, between Toulon and Nice). Investigated by the same complementary approaches, these samples proved to be two new species of blue diatoms. They were named *Haslea silbo* sp. inedit. and *Haslea provincialis* sp. inedit., respectively [21]. They are currently being characterized. Both species produce a blue pigment, which is highly similar if not identical to marennine, as evidenced by UV-visible spectrophotometry (not shown), and Raman spectrometry (Figure 4). Both techniques did not allow discriminating between intracellular and extracellular forms of these pigments, in contrast to marennine. However, these two techniques could be less suited for going further in the study of these pigments. For instance, Raman spectroscopy showed that the pigment purified from a supernatant (extracellular form) of *H. provincialis* sp. inedit. displays a spectral signature similar to IMn, but the NMR spectra appear to be more distinctive. Figure 5 compares the ^1^H NMR spectra of the extracellular form of the pigment from *H. provincialis* sp. inedit. with EMn and IMn from *H. ostrearia*. All three spectra show a general similarity in the represented signal groups, indicating that the molecules belong to the same substance class. There is essentially an important signal group at 3.4 to 5.4 ppm, assigned to sugar ring hydrogens, and the signals in the aliphatic region between 0.8 and 2.4 ppm (for a more detailed description of the NMR studies see paragraph 4.3). There are only weak signals in the aromatic region. However, there are differences in the details. Most strikingly, both extracellular forms contain a strong CH_2_ signal at 1.22 ppm. Furthermore, the intracellular form of marennine appears to contain less anomeric protons in alpha conformation (4.9–5.7 ppm). Overall, from what can be seen in simple ^1^H NMR spectra, the difference between the two extracellular forms appears smaller than the difference between EMn and IMn of *H. ostrearia*. These preliminary results appeal for a more thorough study of the marennine-like pigments produced by the different species of blue diatoms, which constitute an original family of natural blue pigments.

## 3. Mass Production and Methods of Purification

### 3.1. Overview of Former Attempts and State of the Art

A series of engineering- and process-oriented studies were conducted at the end of the 1990s dealing with biotechnology issues of *H. ostrearia* production, in addition to marennine release and extraction with photobioreactors and membrane separations [61,62]. Vandanjon *et al*. [63] proposed a method to produce a large amount of extracellular marennine (EMn) by simultaneous concentration and desalting of the pigment released into the culture medium (Blue Water) using a 3 kDa cut-off ultrafiltration membrane. The resulting permeate contained EMn and a mixture of several components in a large range of molecular weights, as only a 3 kDa cut-off membrane was used. This experimental approach therefore did not allow the obtaining of suitably pure pigment. On the other hand, the aim of this work was mainly to concentrate a large amount of marennine for developing potential industrial applications, which did not require an absolutely pure product. 

Subsequently, in order to increase the recovery of the intracellular marennine (IMn), a continuous-flow-high-pressure homogenizer was evaluated [64]. Cells were partly broken from 30 MPa, but a pressure of 100 MPa (1 cycle) was required to obtain optimal pigment release. The latter was directly linked to the physical cell breakage dependent upon the applied pressure and the number of disintegration cycles. Granulometric analyses by laser diffraction (0.04–2000 μm) revealed a size reduction of cell fragments when increasing these two operating parameters. 

In view of optimizing both production rates in photobioreactors and extraction yields of marennine, Vandanjon *et al.* [63] studied the effects of shear stress on *H. ostrearia* cells due to circulation in pumps and valves of the production or harvesting systems. For the pumps, it was shown that shear stress was dependent on the type of pump, but that mechanical shear could have different effects even if the pumps and the number or frequency of loops were the same. In throttling valves, the aim was to correlate the effect of shear to a parameter related to the inner geometry of the valve and to operating conditions. An overall parameter was then evaluated, *i.e*., the pressure drop coefficient *Kv* that integrates both the type of valve and its opening degree. As a consequence, the modeling of the shear effects was conceivable; basic descriptive data used so far (type of pump, geometry or opening degree of the valve, *etc.*) were completed and partially substituted by quantitative parameters (rotating velocity, capacity, or internal leakage for the pumps, *Kv* coefficient for the valves). 

A new photobioreactor coupled with an ultrafiltration system (immersed membranes) was investigated for the continuous culture of *H. ostrearia* in order to improve marennine production and recovery [65]. The system, with a simple design, was particularly interesting, because energetic costs were minimized, and the cells were not submitted to any shear stress due to pumping or circulation. The photobioreactor was of the cylindrical type; a membrane module was placed at the bottom of the reactor and the hydrostatic pressure was used as a driving force both for the permeation and periodical backflushing steps. The production of biomass and marennine was stable for a three-week period, with marennine concentration three times higher than in a conventional batch photobioreactor. 

A final study dedicated to bioprocessing aimed to compare the pigment productivity obtained with two types of photobioreactors [66]. In the first process, cells were free and recycled in a photobioreactor combined with membrane ultrafiltration equipment (external loop). In the second system, cells were entrapped in a tubular agar gel layer in a photobioreactor of original design. The influence of nitrate concentration and renewal rate was examined. Experiments, conducted over long-term periods (up to 40 days) without any external contamination revealed that marennine productivities of more than 5–7 mg 10^9^ cell^−1^ day^−1^ could be reached with both bioreactors. The advantages and drawbacks of each process design were also discussed. 

**Figure 6:**
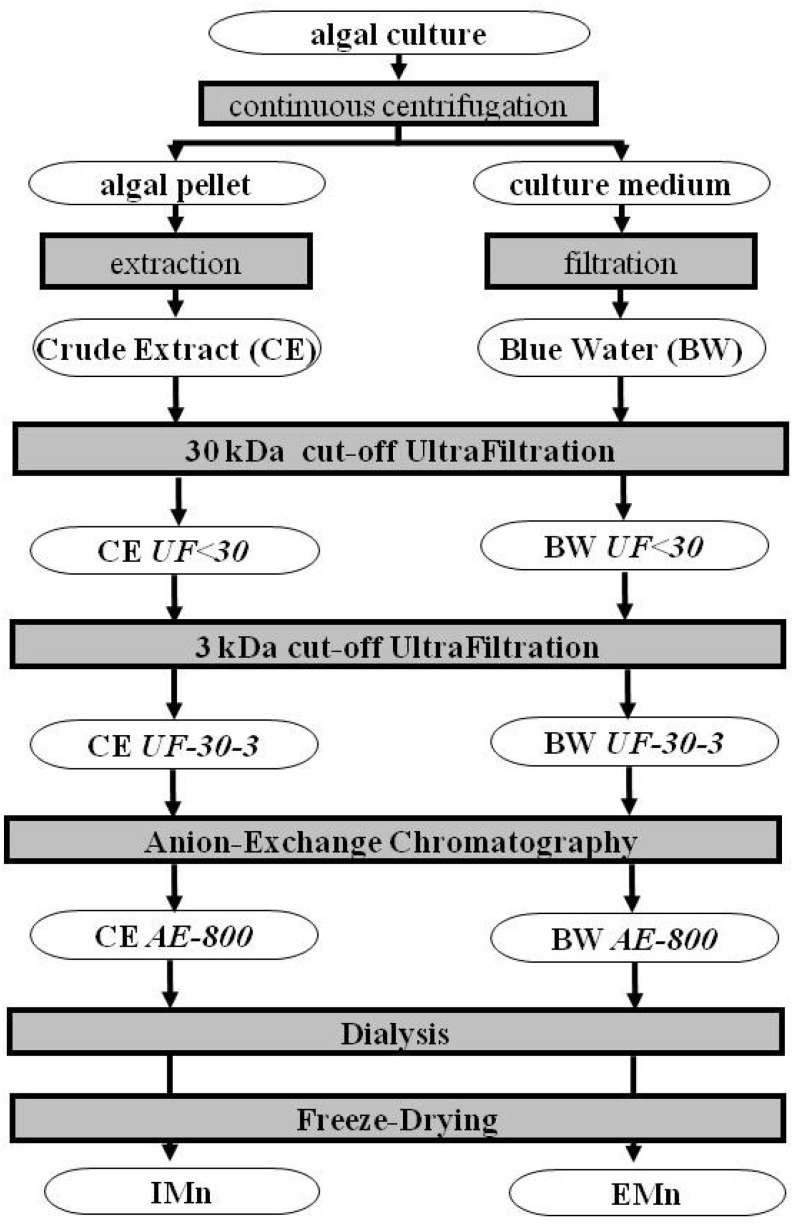
Purification process of marennine and marennine-like pigments.

Marennine is insoluble in all organic solvents, and first attempts to extract it from algal biomass were made using various aqueous solvents, e.g., pure distilled or tap water, or bicarbonate or phosphate buffers. Regarding purification, Robert *et al*. [19] published a purification procedure in which intracellular marennine (IMn) was extracted using organic solvent and water mixtures and further solubilized in 500 mmol L^−1^ K_2_SO_4_ at 80 °C. This method obviously increased the possibility of obtaining denatured pigment and was not convenient to produce large amounts of IMn. More recently, Pouvreau *et al.* [67] developed a new method for the extraction and purification of both forms of marennine (Figure 6). EMn and IMn were selectively extracted from Blue Water (blue-colored culture medium) and algal pellet, respectively, and were then purified by a three-step semi-preparative procedure using two ultrafiltration steps and one anion-exchange chromatography step. This method was easily applicable to a large production system. Finally, after dialysis, UV-visible-photodiode array analysis showed that EMn and IMn were not contaminated, suggesting that these compounds reached the degree of purity required for further biochemical investigations.

### 3.2. Recent Transfers to a Pre-Industrial Scale

Fundamental research regarding the characterization of marennine-like pigments can be sustained by algal production at the laboratory scale, but mass production of *H. ostrearia* is a prerequisite before achieving any added value at the industrial scale. Therefore different attempts were made to grow this peculiar diatom. According to the literature, *H. ostrearia* has been cultivated mostly at the laboratory scale (<100 L), but also at a semi-pilot scale (up to 10 m^3^) [68], or using photobioreactors (<7 L) [65]. At a larger scale, *H. ostrearia* was grown industrially for several years under indoor controlled conditions to produce marennine, mainly for the intensive greening of oysters (knowledge transfer from U. de Nantes to SOPROMA, Bouin, France); however, this production unit ceased its activity about seven years ago due to lack of funding. In fact, the only structures for the mass production of *H. ostrearia* that have ever been fully operational are oyster ponds. Unfortunately, blooms of *H. ostrearia* in these ponds (and as a consequence the greening of oysters) remain erratic and non-controllable. Production units of *H. ostrearia* still need to be developed, taking into account the specificities of benthic microalgae, that use tank surface to grow and generally tend to form biofilm, which makes them difficult to grow in conventional culture systems designed for suspended microalgae. Since the technology to produce massive algal biofilms is not readily available, a simple photobioreactor could be designed in order to maximize *H. ostrearia* productivity and, mostly, excretion of marennine. Some relevant traits of *H. ostrearia* autecology must be considered to design the culture system: growth is characterized by a settlement on the marine sediment, where maximal biomass (as far as 350,000 cells L^−1^) is obtained in conditions of shallow waters (as in the oyster ponds) and high light intensity [47]. Marennine is then released in the water, with concentration ranging between 2 and 5 mg L^−1^ [68]. 

With this goal—but also these constraints in mind—a photobioreactor (PBR) dedicated to the production of *H. ostrearia* and marennine has been devised. This simple PBR was designed in order to obtain large quantities of marennine to further study its biological activities and chemical structure. Also, it was designed in a simple manner so that it could be easily setup by end-users, in laboratories as well as in hatcheries. Flat-bottom polyethylene circular tanks of 200 L were used (diameter: 122 cm), covered by acrylic glass with light supplied from above by T5-5000K high-output fluorescent tubes (General Electric, Mississauga, Canada) at about 25 cm from the cover. A drain was installed at the lowest point of the tank, allowing water sampling and harvesting. Pre-filtered air (0.22 μm) was supplied to the dead space inside the tank through the cover to keep a positive pressure inside the tank, preventing contamination from the outside and purging air heated by the proximity of the light source. The proximity of the air source relative to the water surface also generated a slight agitation at the surface, thus ensuring a slow but constant water movement in the tank and providing nutrient renewal to the cells. 

**Figure 7:**
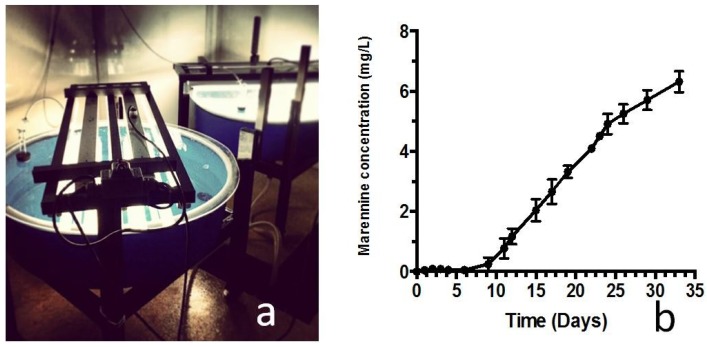
(**a**) Photography of the two PBRs used in this experiment; (**b**) Marennine concentration (mg L^−1^) in the PBR, measured spectrophotometrically on the culture medium. Values are means of concentrations obtained in each PBR (*n* = 2).

The study was conducted at the Université du Québec à Rimouski (UQAR) Station Aquicole at Pointe-au-Père (Québec, Canada), with the use of two PBRs. Algal production was carried out using the NCC-136 strain of *H. ostrearia* isolated from Bourgneuf Bay (Bouin, France) and provided by the Nantes Culture Collection (NCC). Cells were grown in a semi-continuous mode in sterilized seawater enriched with F/2 [69] and 30 mg L^−1^ silicates. Culture was initiated in 500 mL Erlenmeyer flasks filled with 200 mL seawater, and then transferred to 2.8 L Erlenmeyer flasks filled with 2 L seawater. Growth irradiance was 125 μmol photons m^−2^ s^−1^ and room temperature was maintained at 20 °C. Two of these 2 L flasks were then used to inoculate one PBR with approximately 2000 cells mL^−1^. To minimize light attenuation by the water column (<10 cm), tanks were half filled with 100 L filtered (1 μm) natural seawater (salinity 28) supplemented with commercial nutrients (f/2 and silicate from Fritz) and ultrafiltered at 50 kDa (Romicon, KOCH Membrane, Wilmington, MA, USA). The irradiance level was 180 μmol photons m^−2^ s^−1^ (PAR Radiometer, Q201, Macam photometrics LTD., Livingston, Scotland, UK), measured under the light source at the bottom of the empty tank. Light intensity decreased in a linear way to reach 100 μmol photons m^−2^ s^−1^ at the border of the PBR (Figure 7a). Room temperature was kept at 16 °C by an air-conditioner, for a maximal water temperature of 19.5 °C. Both rooms were kept in a 14/10 h light/dark cycle. Two cultures were run in separate PBRs, under the same conditions. Marennine concentration was determined on the cell-free culture water (syringe-filtered on 0.22 μm) by optical absorption according to the Beer-Lambert law. Optical density (OD) was measured at 677 nm in a 10 cm cell by means of a Cary 100 Bio UV-Visible spectrophotometer (Agilent Technologies, Mississauga, Ontario, Canada), using the specific extinction coefficient for EMn following Pouvreau *et al*. [70]. Optimal algal biomasses in the tanks, based on cells and biofilm appearance, were obtained after 12 days of culture growth, as with pre-culture realized in smaller volumes (confirmed with Nageotte counting chambers). Marennine release into the medium started after 10 days of growth (Figure 7b), which corresponds to the end of the exponential phase. Marennine production was relatively constant between day 10 and day 33, with a production rate of about 0.3 mg L^−1^ day^−1^. Maximal extracellular concentration obtained was about 6.2 mg L^−1^ in both PBRs. 

Different culture methods were tried in the past by different authors, with estimated marennine productivities ranging from 3 to 100 mg L^−1^ [65,66,68,71,72,73,74], but these values were obtained using a cytophotometric method and a calibration curve established with unpurified marennine, and not referring to a published extinction coefficient [75], which is more an estimation of marennine production in a culture rather than a true quantification of the pigment [70]. In the literature, there is no quantification of marennine production in natural environments or in a culture that uses the molar extinction coefficient determined on purified marennine by Pouvreau *et al*. [70]. Therefore, a direct comparison of our results with other results in the literature is difficult. 

## 4. Historical Perspective on Marennine Studies

### 4.1. Biosynthesis of Marennine: When, Where, Why, and How It Happens

Although the work of Pouvreau *et al.* [70] represents the most advanced investigation on marennine characterization thus far, the exact structure of marennine is still unknown, and the description of its biosynthesis pathway still a distant goal. It is also unclear when marennine synthesis is triggered in the cell. There is consensus on the stimulating effect of high light conditions and long photoperiods [49,76,77] with just a few authors advocating the opposite [78]. An impact of the light quality, with a positive effect of blue wavelength, has been documented [19,76,77]. Some authors suggested that marennine production could be influenced by organic substances like glucosamines [78,79], or result from salt-induced stress [32,49,80,81], or nutrient deficiencies, e.g., iron, vitamins [47,49]. In the line of the “nutrient deficiency hypothesis,” it has also been suggested that marennine synthesis could result from, or be enhanced by, the senescence of cells [82]. It is worth noting that this could be the result of an apparent and transient accumulation inside the cells, due to a reduced division rate, rather than an increase in production yield. Furthermore, it has been shown that *H. ostrearia* cultures in the exponential growth phase release marennine continuously in the medium [19], but the mechanisms responsible for the release of marennine out of the cell and the factors that control this release are not yet determined. However, according to Nassiri *et al.* [83], marennine is accumulated in vesicles at the apex of the cells and excreted by exocytosis. A type of secretory vesicles containing marennine (a few μm in diameter) can be observed outside the plasmalemma in contact with cells [83]. These vesicles attach to the cells during their erratic movements, then they detach from the cells, still float for a few minutes, before they finally collapse and release marennine in the medium. 

There is no consensus on a possible relation between marennine production and photosynthetic pigment content, some authors having observed a negative correlation [32,49,78,84,85], others no correlation [19,47,86,87]. A striking feature associated with marennine production and accumulation in the cell apices is the reduction in size of chloroplasts [32,49,55,78,88,89,90,91,92,93], possibly related with a decrease in photosynthetic activity [86]. However, the plastid size change could also reflect the decrease of the cellular content of major photosynthetic pigments (chlorophylls *a* and *c*, fucoxanthin), when growth irradiance increases [51], a common photoacclimation response in phytoplankton. Moreover, the impact of this size modification on the organization and stability of thylakoids remains uncertain, as observations were contradictory [83,85]. 

Regarding the autecology of *H. ostrearia* in oyster ponds, a few authors hypothesized a correlation between the accumulation of marennine in the cells and a change in algal behavior, switching from the planktonic to the benthic stage, together with changes in its metabolism [47,78,79], blue cells of *H. ostrearia* becoming able to assimilate reduced forms of nitrogen and carbon [52]. Moreover, the many biological activities displayed by marennine and marennine-like pigments (see below) could explain why these specific pigments represent a competitive advantage for the blue diatoms.

### 4.2. The Various Hypotheses Regarding the Chemical Nature of Marennine

For more than hundred years, the presence of such intriguing and unusual blue pigment inside unicellular algae has been a fruitful matter for contradictory theories and inconclusive experimentation. The hypothesis of a metallic salt was proposed early and rapidly rejected [22,26,35,36,94]. Then there was the “pigment connection”, with hypotheses successively relating marennine to carotenoids [79], or to chlorophylls [32,47,55,80,81,85], possibly resulting from their degradation. Other authors linked marennine with cyanobacterial pigments, thus suggesting a protein nature for this water soluble molecule [54,75,95] or proposed it could be an anthocyanin, with respect to stress and pigment accumulation in cells [96]. Finally, recent advances related marennine to a mixture of different macromolecules [97], or to a polymer possibly of polyphenolic nature [70]. It has been shown that marennine exists in two slightly different forms, intracellular and extracellular [67], and that for both forms, the color changes with pH from blue (acidic pH) to green (basic pH) [70].

### 4.3. Recent Discoveries on Marennine’s Structure

Following the preliminary characterization by Pouvreau *et al.* [70], a series of experiments are currently being conducted to extend our knowledge about the chemical structure of marennine, mainly using nuclear magnetic resonance (NMR) techniques. All current NMR experiments have been carried out on a Bruker Avance 400 MHz spectrometer equipped with a 5 mm BBFO^+^ probehead. Samples of EMn, purified as described in Pouvreau *et al.* [67] were dissolved in 0.5 mL of 25 mM phosphate buffer in D_2_O with 40 mM NaN_3_ at pD 6.6 (corresponding to pH 7.0) to a concentration of 2.4 mM. A ^1^H–^13^C HSQC was acquired with 64 repetitions and 800 increments in the indirect dimension, applying the echo–antiecho scheme, until a maximal *t*_1_ of 24.8 ms. A ^1^H TOCSY with 90 ms DIPSI-2 mixing was acquired using 16 repetitions and 800 increments in the indirect dimension until a maximal *t*_1_ of 106 ms. 

**Figure 8:**
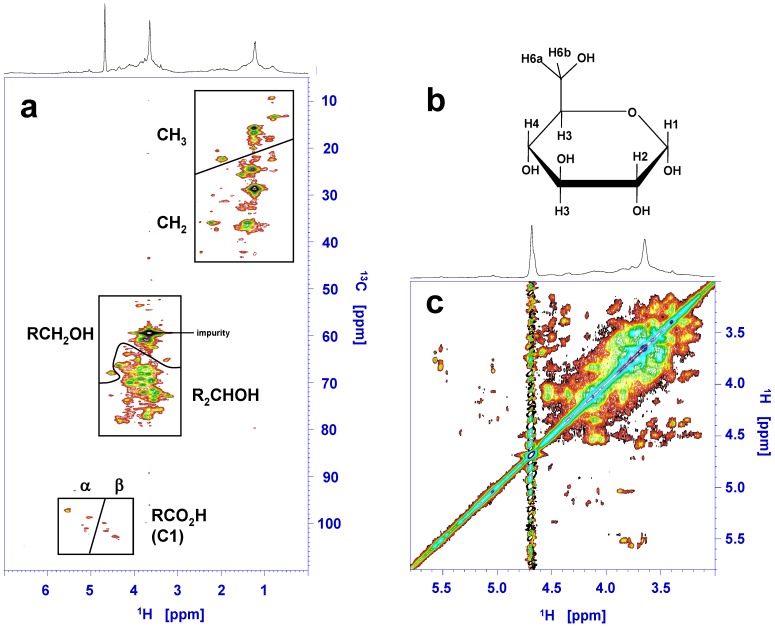
[2DNMR]. (**a**) ^1^H–^13^C HSQC of the extracellular form of marennine (EMn) in deuterated phosphate buffer. On top the corresponding 1D ^1^H spectrum. The lines separate areas of carbons with—among others—two hydrogen atoms and with one or three hydrogens, as determined by an additional edited HSQC with lower resolution and signal-to-noise ratio; (**b**) Generic pyranose; (**c**) Ring region of a ^1^H–^1^H TOCSY of EMn with 90 ms mixing time. The strong ^1^H signal at 3.65 ppm stems from an impurity.

The standard one-dimensional ^1^H spectrum of EMn shows signal groups without any resolved individual signals (Figure 5 and top of Figure 8a). Marennine is, therefore, a macromolecule with a relatively complex structure. ^1^H diffusion spectra (“DOSY”) reveal a diffusion coefficient of about 10^−10^ m^2^/s for both EMn and IMn, consistent with the mass in the 10 kDa range determined by mass spectrometry [70]. The high signal at 3.58 ppm is identified as impurity. 

A (partial) resolution of signals can only be achieved by two-dimensional [2D] NMR. The correlation signals in a ^1^H–^13^C HSQC (Heteronuclear Single Quantum Coherence) spectrum identify chemical groups by a combination of the hydrogen and carbon chemical shifts. An edited HSQC enables, in addition, the determining of the number of hydrogen atoms attached to a carbon by the sign of the signal. The sensitivity is, however, lower. We have acquired an HSQC (Figure 8a) and an edited HSQC of EMn (not shown). Three groups of signals can be seen in the HSQC spectrum, the most striking being in the chemical shift ranges of (60–75 ppm) × (3.5–4.5 ppm). This chemical shift distribution matches—among others—the ring carbons (C2–C5 in the case of a pyranose) and hydrogens of saccharides. This is supported by a small group of signals in the relatively unusual chemical shift region of (95–105 ppm) × (4.3–5.8 ppm), characteristic of anomeric carbons of aldoses (C1) and their hydrogens. According to the edited HSQC, these signals belong to a carbon bound to—among others—one hydrogen atom. Only some signals with relatively small carbon shifts stem from carbons bound to two hydrogens, corresponding to a CH_2_OH group (C6; also C1 in some furanoses). According to the chemical shifts of both hydrogen and carbon atoms, the anomeric signals can further be divided into a group attributable to carbohydrates in the α (97–102 ppm) × (4.9–5.7 ppm) and the β form (usually 103–106 ppm, here lower) × (4.3–4.8 ppm). The interpretation of the HSQC spectrum is confirmed by ^1^H–^1^H correlation experiments. Figure 8b displays the ring region of the TOCSY (Total Correlation SpectroscopY) spectrum. Typical for polysaccharides, there is much spectral overlap in the region of cross peaks between the non-anomeric ring protons by a high density of signals with little dispersion (Figure 8c). Cross-peaks of H2 to α-anomeric hydrogen signals H1 are faint even after relatively long mixing time (90ms), because the equatorial–axial *J*-coupling is weak [98].

The fact that marennine consists to a large extent of glycosidic elements suggests a connection to the main energy storage compound of diatoms, chrysolaminarin, in particular under consideration of the large amount of marennine produced by *H. ostrearia*. The spectra are, however, more complex than those of chrysolaminarin [99]. Moreover, there is a large number of signals in the aliphatic region at about (10–40 ppm) × (1.0–2.5 ppm). The CH_3_ and CH_2_ areas are separated by their chemical shifts and the edited version of the HSQC. A particularly high ^13^CH_2_ signal has a chemical shift of 28 ppm; it is already clearly visible in the 1D ^1^H spectrum at 1.22 ppm. Diffusion experiments (Diffusion Ordered SpectroscopY, DOSY) confirm that this signal comes from the macromolecule and not from an impurity. It could indicate the presence of longer CH_2_ chains. Such a unit would, however, conflict with the general hydrophilic character of marennine. Steroids are an aglycone substance group that could explain the general dispersion of aliphatic signals. 

In a previous study using primarily biochemical tests on various potential substance groups [70], a glycosidic digestion test on sugars had been negative. It is possible, however, that the other chemical groups compromised the detection by this method. On the other hand, it was concluded that marennine was a substance with polyphenolic or similar rings, but signals from the aromatic region in the HSQC spectrum are too weak to detect any correlation under the present experimental conditions (see also Section 5). The Folin-Ciocalteu and the Prussian Blue tests on polyphenols might have been positive due to these few groups, while NMR identifies the scaffold of the macromolecule. This also counts for the chromophore. Whatever its nature is, polyphenolic or of a different type, it is very possible that it constitutes only a small part of the molecule, which does not become apparent from the first impression from the principal NMR signals of this molecule. Pouvreau *et al.* [70] also report the elemental composition of marennine, where, remarkably, oxygen constitutes about 50% of the molecular mass. This result—since then confirmed using different samples of purified marennine—is consistent with a polyphenol but also with a glycoside. 

## 5. Marennine Possible Functions and Biological Activities

During a bloom of *H. ostrearia* in an oyster pond, the quantity of marennine released into the environment should represent a high “cost of production”, which raises a question about the advantages that the species may derive from this peculiar biosynthesis pathway. More generally, the ecological significance of marennine-like pigments remains to be considered, especially in regards to the amount of pigment produced during the diatom life cycle. As presented above, historically, a wealth of information is available on marennine, but concerning the diatom *H. ostrearia* itself, information about the biological function of this specific blue pigment is scarce and inconclusive. The longest known and most obvious biological effect of marennine is the greening of oysters. Experimentally, oysters placed in either a suspension of *H. ostrearia* or a green supernatant of a culture, turn green within a few hours [55,57,82,89,100,101], but little is known about the mechanism. Some authors suggested there might be an interaction between marennine and some proteins in the gills [36], especially in some specialized secreting cells [22,36]. In oyster gills *in vivo*, it is possible that marennine binds and precipitates proteins like tannins [60]. A tannin-binding protein effect would be in agreement with the suggested polyphenolic nature of marennine [70]. Regarding *H. ostrearia* itself, Schubert *et al*. [102] showed that marennine did not play any role in light capture and photochemical activity; however, it could indirectly influence photosynthesis by absorbing in the red part of the spectrum, with peaks at 672 and 677 nm for the intracellular and the extracellular forms of the purified pigment (neutral pH), respectively [70], and at 669 nm for raw supernatants of *H. ostrearia* cultures [19]. Marennine could thus be considered a photoprotective molecule at high irradiance levels [103], or a factor able to modify the light spectrum in the water column when accumulated in the medium [70,77].

Apart from this shading effect, it was shown that marennine has antioxidant activity [103], that it can afford some protection against metals such as copper [104], and act directly as an allelochemical by inhibiting the growth of some algal species encountered in oyster ponds and modifying inter-specific competition among phytoplankton [49,105]. These results have been reinforced by co-cultivation experiments of *H. ostrearia* with other species, which underlined the sensitivity of centric species like *Skeletonema costatum*, *Chaetoceros calcitrans*, *C. gracilis*—all species abundantly used in aquaculture—as well as the insensitivity of others like *Pavlova lutheri* (not shown). This could explain the occasional dominance of *H. ostrearia* in oyster ponds, concomitant with an almost complete elimination of other diatom species [47,68,79], thus revealing the importance of chemical ecology in marine phytoplankton and environments [106]. A few authors hypothesized that *H. ostrearia* could itself be affected by marennine, a sort of autotoxin associated with pathological processes [32,49,79]. However, it was further demonstrated that *H. ostrearia* was rather tolerant to marennine [70].

Last but not least, preliminary works conducted on *H. ostrearia* aqueous extracts, thus containing marennine, displayed antiviral, anticoagulant [107] or growth-inhibiting properties [108]. These activities have been recently confirmed using purified marennine, which exhibited antibacteria, antivirus, and antiproliferative activites [60], or using the pigment synthesized by *H. karadagensis*, the pigment of which demonstrated antifungi, antibacteria, and antiviral activities [57]. Hence, aside from the greening action, marennine and marennine-like pigments could be especially useful in prophylaxis in the context of oyster farming, due to their antibacterial and antiviral activities.

In the last decades, it has been observed that the cultivated Pacific oyster *Crassostrea gigas* presents massive and recurrent summer mortalities, this being of great concern for the oyster industry all over the world. In France, for instance, bacteria such as *Vibrio aestuarianus* [109], *V. splendidus* [110], and viruses belonging to the Malacoherpesviridae, like the ostreid herpesvirus OsHV-1, distantly related to other members of the Herpesviridae [111] are frequently associated to, if not responsible for, these severe summer mortality events. 

Marennine-like pigments displayed antibacterial activities against three marine bacteria, *V. aestuarianus*, *Pseudoalteromonas elyakowii*, *Polaribacter irgensii* [57,60]. In a first attempt to explain the mechanism of action of the antibacterial activity exhibited by marennine-like pigments, Tardy-Laporte *et al*. [112] demonstrated, using ^2^H solid-state NMR on intact *Escherichia coli*, that the extracellular form of the pigment produced by *H. provincialis* sp. inedit. disturbs the bacteria membranes, unlike the intracellular form. More specifically, their results suggest that the pigment released in the medium exerts its antibiotic action by interacting with the lipopolysaccharides on the bacterium’s surface, thus rigidifying the outer membrane. 

**Figure 9:**
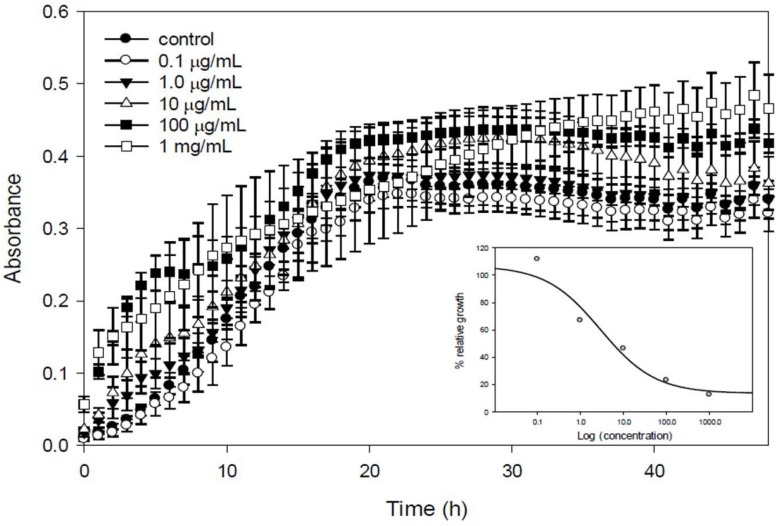
Growth of *Vibrio splendidus* after 3 h in contact with marennine. *V. splendidus* was grown in modified marine media overnight. Cells were washed and then incubated for 3 h in 0, 0.1, 1.0, 10, 100, or 1000 μg mL^−1^ marennine. Cells were washed in sterile water and brought to an optical density of 0.5 before the 3 h incubation. Cells were then added to fresh marine media in a 96 well plate and growth kinetics were done for 48 h with measurements every 30 min. Inset: maximum growth rates expressed in function of the control (100%) with marennine concentrations.

Along the lines of this membrane-mediated inhibition effect, a series of experiments were conducted with *V. splendidus* cells incubated for 3 h with different concentrations of EMn (0.1 μg mL^−1^ to 1.0 mg mL^−1^), then washed and resuspended in fresh marine medium, for 48 h. The conclusion was that the higher the marennine concentration, the higher the inhibition of the growth would be (Figure 9). The effective concentration reducing bacteria growth rate by 50%, EC_50_, was 2.89 μg mL^−1^, a value in the range of many anti-bacterial marine compounds described thus far [113].

Regarding antiviral activities, due to a lack of a suitable bivalve cell line for culturing oyster herpes virus, a heterologous model using fibroblastic Vero cells and human HSV-1 was chosen in some studies, to investigate the effect of antiviral activity [114]. It has been shown that marennine intracellular and extracellular forms exhibited antiviral activity against the HSV-1 *herpes* virus [60], with 50% antiviral effective concentration (EC_50_) values of 24.0 and 27.0 μg mL^−1^, respectively (for a multiplicity of infection (MOI) of 0.001 ID_50_/cells). The blue-grey pigment produced by *H. karadagensis* presents similar antiviral activity [98], with the extracellular form being more active than the intracellular form (EC_50_ of 23 μg mL^−1^ and 62 μg mL^−1^, respectively). Both intracellular forms of the pigments present a relative cytotoxicity against the fibroblastic Vero cells, not observed with their extracellular counterparts. This underlines the need for further experiments regarding a possible toxicity, and the mode of action of marennine-like pigments. A sulfated polysaccharide, naviculan, was isolated from *Navicula directa*, a diatom collected from deep-sea water in Toyama Bay, Japan [115]. This compound was shown to inhibit HSV-1 and HSV-2 (half maximal inhibitory concentration, IC_50_ = 7–14 μg mL^−1^) by interfering with the early stages of viral replication, most likely affecting viral adhesion and penetration into host cells. Very few other biologically active secondary metabolites have been reported from diatoms [116,117], and except for a few studies [57,60,107], little is known about the antiviral activity of diatom extracts or compounds. The selection of antiviral marine compounds for aquaculture environments being a possible solution to control viral disease transmission [118], a possible valorization of the biological activities of marennine-like pigments, would constitute a new and promising field of research. 

The fact that marennine-like pigments seem to have the potential to inhibit growth of pathogenic marine bacteria and virus replication, is of special interest for oyster producers, as bivalves lack antibody-mediated humoral immunity, and possess instead an innate immune system. Experiments are in progress to test the hypothesis that marennine in solution or present on gills could protect oysters from these pathogens in realistic *in vivo* conditions. 

## 6. The Putative Commercial Potential of Marennine-Like Pigments

At the industrial scale, marennine is solely exploited in aquaculture for the greening of oysters, thus as a natural blue-green colorant. The main source of natural blue colorants is indigo dye, extracted from different species of higher plants. For the food industry, however, there is a lack of available natural blue pigment alternatives compared to red, orange, and yellow natural colorants [119]. More simple to produce and less expensive to use, some of the synthetic blue colorants used in food chemistry have to face alleged or assessed reputations of harmfulness, some of them possibly encountering commercial restrictions or banishment (e.g., Patent Blue V E131). Presently, the use of marine blue dye in the food industry is restricted to phycocyanin, mainly produced by cyanobacteria but also some Rhodophyta [120]. Phycocyanin is a food colorant, which is known in Japan under the name of “Lina-Blue”, and it is mainly used in ice-creams or drink preparations. In this context, marennine is also a natural blue pigment, which could complement the source of marine blue dyes available for the food industry, and it presents some merits but also a few flaws. People have consumed green oysters for centuries, without any disease or anaphylactic reactions recorded, which should suggest that this pigment is non-toxic, when considering a standard food intake. Marennine is produced by a marine microalga, which augurs well for an environmental-friendly production system. It is water soluble, and its extraction process does not require massive use of solvents. However, the structure of marennine is still unknown, and because marennine exhibits a wide range of biological activities, its possible cytotoxicity and its stability as a pigment have to be carefully tested before considering it for possible use in cosmetics. Indeed, before turning into a reality in Europe, and in France especially, this type of application will need to be submitted for approval. For example, the use of “pure marennine” as a food colorant would indeed require nutritional and toxicity studies on rats, and cellular tests to demonstrate that the pure molecule has no mutagenic properties. These necessary steps represent an immense obstacle to using marennine as a food additive. An alternative method, already applied for the upgrading of phycocyanin and phycoerythrin, would be to promote the use of aqueous extracts enriched in pigment for their colorant properties. Generally, this application is authorized, at least for phycocyanin, and appears to be realistic from an economic point of view regarding cosmetics; however, this would become an even more crippling problem for any development in the food and health industry.

Illustrating the added-value potential of some microalgae in cosmetic industry, a few companies such as LVMH group and Daniel Jouvance have invested in their own microalgal production units. Indeed, some microalgae (e.g., *Chlorella*, *Odontella*, *Tetraselmis*, *Dunaliella*, *Emiliania*, *Noctiluca*) are established in the skincare market. Microalgal extracts are mainly found as skincare products, e.g., anti-aging, emollient or moisturizing, and also sunscreen products. Preliminary experiments were conducted to study the possible photo-protective and anti-inflammatory effects of marennine. The photo-protective potential was thus studied, to determine sun protection factor (SFP) and UVA protection factor (PF-UVA) of marennine, using an *in vitro* method. An O/W emulsion placebo was prepared in the laboratory as previously described [121]. Marennine was incorporated into the formulation components at 10% (w/w) in order to study the potential biological properties. Thirty milligrams of product exactly weighed were spread on polymethylmetacrylate (PMMA) plates over the whole surface (25 cm^2^) using a cot-coated finger (15 mg remain on the finger cot). SPF and PF-UVA of the creams were measured *in vitro* [122,123]. Three plates were prepared for each product to be tested and nine measurements were performed on each plate. Transmission measurements between 320 and 400 nm were carried out using a spectrophotometer equipped with an integrating sphere (UV Transmittance Analyzer UV1000S, Labsphere, North Sutton, NH, USA). Emulsions containing 10% (w/w) marennine exhibited SPF (1.28 ± 0.05) and PF-UVA (1.24 ± 0.04) values (means ± SD, *n* = 3), which demonstrate that marennine could not be considered a molecule interesting enough in the domain of the topical photo-protection.

The determination of a possible anti-inflammatory effect of marennine incorporated into a cream formulated in the laboratory was carried out using a test with Phorbol-12-Myristate-13-Acetate (PMA). Introduction of mouse ear edema was based on the method of Carlson *et al*. with some modifications [124,125,126]. Firstly, the thickness of the mouse ears was measured using a model micrometer gauge (Oditest^®^, Kroeplin, Schlüchtern, Germany). Ten mL of preparation with marennine or 0.1% (w/w) butyrate hydrocortisone-based lotion (Locoïd^®^, Astellas Pharma—Levallois-Perret, France) were applied using a ripette genix electro dispenser (Fisher Scientific, Illkirch, France), on the mice’s right ears, twice at 5 min intervals. Ten mL of placebo emulsion were applied according to the same protocol, on the mice’s left ears. Thirty minutes later, 10 mL of a hydro-alcoholic solution of Phorbol-12-Myristate-13-Acetate (250 mg/mL) were then applied on each ear, in order to cause an edema. After 3.5 h, the thickness of the ears was once again determined using the Oditest^®^. Five mice were used for each product tested. The cream containing 10% (w/w) marennine demonstrated a moderate anti-inflammatory effect, with an edema inhibition of 62.5%, as compared to 100% for the control butyrate d’hydrocortisone (0.1% w/w). These preliminary experiments show that marennine can hardly be considered as a potential UV filter, but it could be valorized in soothing creams. It might be interesting to explore potential applications in the management of atopic skin or rosacea, for example.

## 7. Conclusions

Aquaculture still represents the most immediate and “natural” valorization approach of marennine and marennine-like pigments, due to the greening of bivalves. Beside this coloring action, it is hypothesized that marennine-like pigments could act as natural prophylactic agents in hatchery and oyster farming, due to their antibacterial and antiviral activities, which were demonstrated *in vitro* at the laboratory scale. Alongside possible usage as natural blue colorants for cosmetics—provided they prove to be inexpensive to produce, stable in the formulations, and safe to use—the many biological activities evidenced thus far represent a new research avenue, and a great potential of valorization for the marennine-like pigments. 
